# The Region-Specific Genetic Relationship and Mutation Characteristics of Viral Hemorrhagic Septicemia Virus

**DOI:** 10.3390/microorganisms14102146

**Published:** 2026-09-23

**Authors:** Zhengmin Lian, Jian Zhang, Changqing Zhou

**Affiliations:** Institute of Livestock, Grass, and Green Agriculture, Gansu Academy of Agricultural Sciences, Lanzhou 730020, China; lzm@gsagr.cn (Z.L.); zhang2024@gsagr.cn (J.Z.)

**Keywords:** viral hemorrhagic septicemia virus, genetic evolution, recombination, region specificity

## Abstract

Since viral hemorrhagic septicemia virus (VHSV) was first identified in rainbow trout, it has evolved into four major genotypes in the process of infecting multiple hosts. The different levels of virulence among genotypes of VHSV are caused by genetic evolution. In this study, the complete genome sequences of global VHSV strains were obtained from the GenBank database and their genetic relationships were analyzed. The maximum likelihood (ML) phylogenetic tree based on the complete genomes of VHSV showed that globally, VHSV strains evolved into two region-specific clades, namely the European clade and the North American clade. North American VSHV strains are more conserved than European strains. Both European and North American strains are subjected to purifying selection. All VHSV proteins are gathered into their region-specific clusters in genetic distance space. Furthermore, North American and Asian strains have organized glycosylation sites in their G protein. An early Japanese strain, KRRV9822, which clusters into the North American clade, has undergone potential recombination. Together, the region-specific genetic relationship and mutation characteristics of global VHSV strains were revealed with a crucial perspective for epidemiological research on VHSV.

## 1. Introduction

Viral hemorrhagic septicemia (VHS) is a significant disease in aquaculture caused by viral hemorrhagic septicemia virus (VHSV). It tends to occur in environments with lower temperatures, particularly in the range of 8–15 °C, with rapid spread among the susceptible fish population and a high mortality rate [1,2]. VHS was originally referred to as Egtved disease and first appeared in Danish rainbow trout around 1950 [3]. VHSV was first isolated and identified in 1962 from diseased rainbow trout farmed in Denmark [4]. It was previously regarded as the specific pathogen of freshwater rainbow trout farmed in Europe until the late 1980s [5]. Subsequently, VHSV infection was reported in multiple freshwater and marine fish species [6], mainly including Japanese flounder (*Paralichthys olivaceus*) [1] and largemouth bass (*Micropterus salmoides*) [7]. Importantly, VHS outbreaks usually cause high mortality rates on fish farms and major economic losses [8,9].

VHSV is one of the highly pathogenic fish rhabdoviruses belonging to the genus Novirhabdovirus of the family Rhabdoviridae [10]. Its genome is a non-segmented and single-stranded negative-sense RNA consisting of approximately 11,000 nucleotides [11]. There are six open reading frames (ORFs) annotated which encode five structural proteins and a non-structural protein including the nucleoprotein (N), the phosphoprotein (P), the matrix protein (M), the glycoprotein (G), the RNA-dependent RNA polymerase (large protein, L), and the non-virion protein (NV) [12,13]. Based on the nucleotide sequences of G and N, VHSV is classified into four principal genotypes (I, II, III, and IV) [14], among which the genotype I can be further divided into six subtypes (Ia to If) [15], and genotype IV can be divided into four subtypes (IVa to IVd) [16,17].

The genotypes I, II, III, and IVd are mainly prevalent in Europe [16], among which genotypes II and III mainly infect wild marine fish in Europe [4,18,19,20]. Genotype I is widely distributed, although the subtype Ia is highly pathogenetic for freshwater farmed rainbow trout in Europe [4,18,21]. In Europe, genotypes I, II, and III prevail in marine-estuarine habitats of the Baltic, North and Black Seas as well as European freshwater aquaculture [22]. It is reported that European isolates exhibit high lethality to freshwater salmonids; the genotypes Ib, II, and III are low pathogenetic or avirulent for rainbow trout [8,19,23]. Additionally, subtype Ia predominantly causes disease in freshwater-farmed fish, whereas subtypes Ib, II and III circulate mainly in marine or estuarine environments [22]. VHSV was introduced into North America in 1988 [24], with subtype IVa circulating in the Northeast Pacific and showing low virulence for marine salmonids [25]. VHSV was detected in Newfoundland (Atlantic Canada) in 1994 [26], and the newly defined subtype IVc occurs in marine-estuarine waters of New Brunswick, Canada [27]. The highly virulent subtype IVb emerged in the Laurentian Great Lakes (shared by the United States and Canada) in 2003 and triggered large-scale epizootics during the 2005–2009 period, infecting 31 fish species before asymptomatic infections became predominant [28,29]. Two separate invasion events took place in the Northwest Pacific (Japan and South Korea): subtype Ib spread eastward from Europe and subtype IVa was transported westward from the North American Pacific coast, both of which can cause mortality in cultured Japanese flounder [30]. A study focusing on the G gene from multiple VHSV outbreaks showed that a main cause of VHSV transmission appears to be movement or fish trade practices [31].

Based on the epidemic characteristics of VHSV, we inferred that the genomes of VHSV strains in different regions might have formed region-specific evolutionary trends. To further reveal the genetic evolution characteristics of the VHSV genome during its global spread, the complete genome sequences of global VHSV strains were obtained from the GenBank database of the National Center for Biotechnology Information (NCBI). Their evolutionary relationship and variation based on their complete genome sequences or single gene were analyzed. Furthermore, the glycosylation sites of G protein from different strains were characterized.

## 2. Materials and Methods

### 2.1. Collection and Preparation of the VHSV Genome Sequences

We downloaded all the complete genome sequences of global VHSV strains (157 strains in total) from the GenBank database of the NCBI (information on the 157 strains is listed in Table S1). The genome sequences were aligned using MAFFT (v7.526) [32]. The coding sequences (CDS) of the N, P, M, G, NV, and L genes were extracted from these VHSV genome sequences.

### 2.2. Establishment of the Maximum Likelihood Phylogenetic Tree

The maximum likelihood phylogenetic tree was reconstructed based on 157 complete genome sequences of VHSV using MEGA12 software. The General Time-Reversible (GTR) nucleotide substitution model was applied, with among-site rate heterogeneity modeled by gamma distribution plus invariant sites (G + I), and five discrete gamma categories were defined. All alignment positions, including gaps and missing data, were retained for phylogenetic analysis. For heuristic tree searching, the Nearest-Neighbor-Interchange (NNI) algorithm was used. The initial ML tree was automatically generated by the default neighbor-joining (NJ) and maximum-parsimony (MP) approaches, and no branch swap filter was implemented. Node support was assessed by standard bootstrap resampling with 1000 replicates to evaluate the reliability of phylogenetic branches.

### 2.3. Nucleotide Homology Analysis

The nucleotide homology of 157 VHSV strains was analyzed via pairwise global sequence alignment using the MegAlign module of DNASTAR Lasergene 7 software. Specifically, the complete genome sequences of VHSV strains were loaded into the MegAlign module, and pairwise nucleotide identity percentages between every two strains were automatically computed. The identity matrix generated using MegAlign was summarized to evaluate the overall genetic similarity degree among the investigated viral strains. Furthermore, the nucleotide homologies of the N, P, M, G, NV, and L genes were computed and evaluated.

### 2.4. Selection Pressure Analysis

For each VHSV gene (N, P, M, G, NV, and L), the CDSs were first translated into amino acid sequences using EMBOSS transeq (v6.6.0). The resulting amino acid sequences were aligned using MAFFT (v7.526) with the auto strategy. The amino acid alignment was then used to guide codon-based alignment of the original nucleotide sequences using pal2nal.pl (v14.1) to preserve the reading frame. For each gene, an ML phylogenetic tree was reconstructed from the codon alignment using IQ-TREE (v3.1.2) with the best-fit substitution model selected by ModelFinder and ultrafast bootstrap approximation.

The ratio of non-synonymous (dN) to synonymous (dS) substitution rates (dN/dS, ω) was estimated for each gene under the one-ratio model (M0) implemented in the codeml program of the PAML package (v4.10). This model assumes a single ω value across all codon sites and phylogenetic branches. All analyses were performed independently for each gene.

### 2.5. Sequence-Based Genetic Distances Analysis

Pairwise genetic distances of VHSV N, P, M, G, NV, and L genes were calculated using the Kimura 2-parameter (K80) model. The resulting genetic distance matrix was used for metric multidimensional scaling (MDS) analysis. The MDS ordination was performed in two dimensions, and model fitting was evaluated using stress values. The MDS results of all strains were visualized as scatter plots to display the genetic clustering and spatial distribution of viral strains.

### 2.6. Analysis of Glycosylation Site in G Protein

The CDSs of the G gene which were extracted from complete genomes of VHSV were translated into protein sequences and loaded into the NetNGlyc web server (https://services.healthtech.dtu.dk/services/NetNGlyc-1.0/, accessed on 5 June 2026). Next, the N-glycosylation sites of the G protein were predicted. All the N-glycosylation sites and their characteristics were summarized, and non-conservative strains were marked in the ML phylogenetic tree of the G gene. The ML phylogenetic tree based on CDSs of the G gene was established with the GTR nucleotide substitution model of MEGA12 software. The heterogeneity across sites was described by gamma distribution plus invariant sites (G + I). All codon positions (1st, 2nd, and 3rd codon positions) were included in the dataset. Bootstrap analysis with 1000 standard replicates was carried out to evaluate the statistical support for each phylogenetic node. Other parameters were kept identical to those used for the complete-genome phylogenetic reconstruction.

### 2.7. Genome Recombination Analysis

The potential recombination events between 157 VHSV genome sequences were scanned via RDP4 software (v4.101) with Bootscan, 3seq, PhylPro, Maxchi, SiScan, and Chimaera algorithms. The major recombination events were further validated using SimPlot software (v3.5.1). In detail, a sliding-window approach was applied with a window size of 500 nucleotides and a step size of 50 nucleotides. To assess statistical support, 100 bootstrap replicates were generated. Genetic distances were calculated using the Kimura 2-parameter substitution model, with a transition/transversion ratio (T/t) of 2.0. Phylogenetic trees were reconstructed using the neighbor-joining method based on the distance matrices.

## 3. Results

### 3.1. VHSV Forms Two Major Region-Specific Clades Based on Complete Genome

The nucleotide sequences of G and N genes are used as a basis for VHSV classification; however, the nucleotide sequences of other genes and the noncoding region in the VHSV genome are associated with their evolution. To analyze the genetic relationship of different VHSV strains, the ML phylogenetic tree was established based on the complete genomes of different VHSV strains isolated from all over the world (information on the VHSV strains used for the ML phylogenetic tree is listed in Table S1). As shown in Figure 1, those VHSV strains form two major clades. The European VHSV strains clustered into a large clade, mainly isolated in Denmark, Sweden, Norway, France, Italy, Finland, Turkey, Georgia, the United Kingdom, the English Channel, the Baltic Sea, and the North Sea. Specially, a Japanese strain isolated in 1996 was clustered into European clade. Additionally, there is a small number of strains isolated from an unknown time or location. In another large clade, the North American and Asian strains are divided into two smaller clades (Figure 1). These strains were mainly isolated in Japan, USA, South Korea, China, and Canada. A small number of North American strains cluster into a branch with Asian strains, and the other North American strains cluster into another branch. It suggested that the Asian VHSV strains have a closer genetic evolutionary relationship with North American VHSV strains. Collectively, the VHSV evolved into two region-specific clades, the European clade and the North American clade (including Asian VHSV strains), based on their complete genome sequences. Based on the strains with known genotypes, the subtypes Ia, Ib, Ic, Id, Ie, and III mainly clustered into the European clade, while the subtypes IVa and IVb mainly clustered into the North American clade (Figure 1).

### 3.2. North American VSHV Strains Are More Conserved

To further assess the nucleic acid differences in these global VHSV strains, nucleotide homology was compared based on their complete genome, N gene, P gene, M gene, G gene, NV gene, and L gene. As shown in Figure 2A, the complete genome of global VHSV strains (157 strains, as shown in Table S1) share 82.3–100% homology, with the majority being less than 87.5%. The N gene, P gene, M gene, G gene, NV gene, and L gene of global VHSV strains share 85.8–100%, 83.3–100%, 85.0–100%, 83.3–100%, 72.1–100%, and 85.1–100%, respectively, with their majority also being less than 90%. In particular, the conservation of the NV gene is the lowest, with the majority being less than 82.5%. In the North America-specific clade (66 strains, as shown in Table S2), their complete genome, N gene, P gene, M gene, G gene, NV gene, and L gene share 92.6–100%, 93.3–100%, 92.8–100%, 94.1–100%, 93.6–100%, 88.1–100%, and 94.1–100%, respectively, with their majority being higher than 97.5% (Figure 2B). In the Europe-specific clade (91 strains, as shown in Table S3), their complete genome, N gene, P gene, M gene, G gene, NV gene, and L gene share 89.7–100%, 91.4–100%, 92.7–100%, 91.3–100%, 94.4–99.9%, 86.2–100%, and 95.3–100%, respectively (Figure 2C). Thus, the genome and individual genes of North American VSHV strains are more conserved than European strains (Figure 2B,C).

### 3.3. VHSV Undergoes Pervasive Purifying Selection

Based on the ML phylogenetic tree and nucleotide homology of different VHSV genes, we inferred that VHSV undergoes different selection pressures between different strains and genes during their evolution. Next, the selection pressure analysis of North American (information on strains is listed in Table S2) and European VHSV strains (information on strains is listed in Table S3) was performed using the CDSs of each gene (N, P, M, G, NV, and L genes). As shown in Figure 3, all calculated nonsynonymous-to-synonymous substitution rate ratios (ω = dN/dS) across genes from both geographic groups were less than 1, ranging from 0.12621 (L) to 0.35208 (NV) for North American VHSV strains and 0.06984 (L) to 0.23328 (NV) for European VHSV strains, demonstrating that purifying selection is the dominant evolutionary force constraining coding sequence evolution for VHSV in both regions. The NV gene coding non-structural protein exhibited the highest ω values in both North American and European strains, indicating the weakest purifying selection. The L gene had the lowest ω values for both North American and European strains, reflecting strong evolutionary conservation under stringent purifying selection.

### 3.4. The Genetic Distances of VHSV Proteins Are Regionally Specific

Although the complete genome of VHSV exhibits region-specific evolution, there are some differences in the nucleotide homology of their different genes. To further analyze the sequence-based genetic distances of different proteins encoded by those VHSV genes, the pairwise genetic distances of N, P, M, G, NV, and L proteins between global VHSV strains were obtained though multidimensional scaling (MDS) analysis based on the K80 model. The results of their distribution patterns showed that the P, M, G, and L proteins form two region-specific clusters in genetic distance space (Figure 4B–D,F). The American (USA and Canada) and Asian (Japan, South Korea, and China) strains are closely gathered together, almost at the same point. In another cluster, the European strains gather in a similar manner. Thus, the genetic distances of the P, M, G, and L proteins are similar to their genomic evolution. For the N protein and non-structural NV protein, all the strains form three clusters. Similar to other proteins, the Asian (Japan, South Korea, and China) strains are gathered together in the genetic distance space of N and NV proteins, and the European strains are gathered into two clusters. Although all the VHSV proteins are gathered into their region-specific clusters in genetic distance space, the N and NV proteins are distributed with a higher dispersion compared with other VHSV proteins.

### 3.5. The Asian VHSV Strains Evaluated a Specific Glycosylation Site in G Protein

The G protein is a glycosylated protein located on the surface of the VHSV envelope [33]. VHSV entry and infection occur though interaction with the cellular receptor [34,35]. To analyze the relationship between glycosylation of G protein and the evolution of VHSV, the potential glycosylation sites of G protein from global VHSV strains were analyzed using the NetNGlyc tool. As shown in Figure 5A, there are seven potential glycosylation sites in the G protein of those global VHSV strains located at 4, 17, 30, 378, 389, 438, and 459 aa. However, only the potential glycosylation sites at 389 and 438 aa of the G protein are conserved in those global VHSV strains, while the other glycosylation sites are not conserved. Furthermore, the distribution of these potential glycosylation sites in different strains was analyzed in an ML phylogenetic tree based on the G gene. The results show that only an Italian strain (MK829678.1, VHSV/O.mykiss/I/BZ/301/Jun00) isolated in the year 2000 has potential glycosylation sites at 4 aa (Figure 5B). There is no potential glycosylation site at the 30th amino acid in the G protein in the Danish F1 strain (MN038327.1, Figure 5B). For the 17th amino acid, there are two Danish strains (MK829411.1 and MK829403.1) and a Swedish strain (MK829415.1) without potential glycosylation sites (Figure 5B). For the 495th amino acid, there are multiple European strains without potential glycosylation sites, mainly including Denmark, Italy, France, and the United Kingdom (Figure 5B). These strains without potential glycosylation sites at 459 aa mainly distribute in the a clade; only a Danish strain is distributed in another clade. In particular, the Asian strains do not have potential glycosylation sites at 459 aa of the G protein, including South Korea, China, and Japan (Figure 5B), and they distribute in a large clade with North American strains. Overall, the prediction results indicate that the potential glycosylation sites of the VHSV G protein are not conserved in European strains with different mutations, and the North American and Asian strains have organized potential glycosylation sites in their G protein.

### 3.6. Recombination Events Occurred in the North American Clade

Although the prevalence of global VHSH strains is region-specific, there are multiple different strains with variant genomes in the same area. To further reveal the evolutionary relationship between different VHSV strains, the genetic recombination events in those VHSV genomes were analyzed using RDP4 software (V4.101). The results of the Bootscan, 3seq, PhylPro, Maxchi, SiScan and Chimaera algorithms show that the two recombination events were only detected in the KRRV9822 strain, and there were no other definite recombination events between other VHSV strains. The KRRV9822 strain is an early Japanese strain which clusters into the North American clade and was isolated from Japanese flounder in 1998. As shown in Table 1, one of the two major recombination events showed that the potential major and minor parental strains are Coho salmon/Washington/W242920039-20/2024 and JF00Ehi1, respectively. Another recombination event showed that the potential major and minor parental strains are KJ2008 and an unknown strain, respectively. Most of the *p* values from the six algorithms in two recombination events were less than 1 × 10^−3^ (Table 1).

Moreover, these potential recombination events were further verified using Simplot 3.5.1 software. The results show that the potential recombination regions in the genome of the KRRV9822 strain are mainly from the Coho salmon/Washington/W242920039-20/2024 strain, which is the latest VHSV strain isolated from American Oncorhynchus kisutch in 2024 (Figure 6). As shown in Figure 6, these potential recombination regions from the Coho salmon/Washington/W242920039-20/2024 strain are mainly located at the 650 to 1100 nt, 2000 to 2650 nt, 4500 to 5000 nt, 5050 to 5800 nt, 5950 to 6450 nt, 6850 to 7400 nt, 8400 to 9300 nt, and 9350 to 9900 nt regions (green regions). In addition, the minor potential recombination regions from JF00Ehi1 and KJ2008 strains are mainly located at the 1100 to 1550 nt, 3450 to 4050 nt, 4050 to 4500 nt, and 7400 to 8400 nt regions.

## 4. Discussion

VHSV is a broad-host pathogen in aquatic animals with strong host adaptability. With the wide spread of VHSV among different hosts and regions, it has been identified to have four genotypes based on the N or G gene [36,37]. To reveal the evolutionary relationship of the VHSV genome, a maximum likelihood phylogenetic tree was established based on the complete genome sequences from 157 global strains in this study. For the reconstruction of the ML tree, the GTR + G + I nucleotide substitution model was implemented. This model is well suited for viral full-length genome sequences, as it accounts for uneven base composition, diverse nucleotide substitution patterns, among-site evolutionary rate heterogeneity, and a fraction of invariant genomic sites. Viral genomes frequently demonstrate insertions, deletions, and partial missing sequences. Therefore, gap and missing data positions were retained rather than trimmed to preserve biologically relevant indel variations that would otherwise be discarded.

Interestingly, two region-specific clades were identified: the European clade (genotypes Ia, Ib, Ic, Id, Ie, and III) and the North American clade (genotypes IVa and IVb). In the North American clade, there are multiple Asian strains isolated from Japan, South Korea, and China. Although these Asian strains are clustered together on the phylogenetic tree, they are close to several North American strains. Thus, these Asian strains were classified into the North American clade. Moreover, the Asian strains and the other three North American strains, which were clustered into a branch with Asian strains, are close to an early strain isolated in 1989 in the USA. Only two VHSV strains were isolated and reported in China [7,38]; their genetic relationships are close to South Korean strains based on their complete genome and genotype (IVa). Thus, we inferred that the Asian VHSV strains might spread from North America and the two Chinese VHSV strains might spread from South Korea. This requires further verification through a more detailed analysis of their genetic relationships, which we will conduct in future research.

There are six ORFs coding five structural proteins and a non-structural protein in the VHSV genome. The nucleotide homology analysis revealed that the genome and individual genes of North American VSHV strains are more conserved than European strains. In addition, the NV gene coding a non-structural protein showed the lowest level of conservation in both North American and European strains. Moreover, selection pressure analysis was performed to further characterize the genetic direction of global VHSV strains. The results reveal that both North American and European strains have undergone strong purifying selection pressure during their evolutionary process. Thus, we inferred that the weak purifying selection of the NV gene led to its low level of conservation, and the differences in European VHSV strains in nucleotide homology were mainly caused by synonymous mutations.

The different evolutions of North American and European VHSV strains implied that there are multiple mutations in their proteins. The genetic distances of individual proteins showed that all the VHSV proteins are gathered into their region-specific clusters in genetic distance space. The surface glycoprotein G is a key target of vaccines and virus-neutralizing agents for fish rhabdovirus. In recent studies, a synthetic hagfish antibody targeting G protein successfully attenuated cytopathic effects in VHSV-infected cell cultures [39]. The rescued VHSV without a full ORF of the G gene could not induce high survival rates through immunization of olive flounder [40]. Moreover, the mutations of potential glycosylation sites in the G protein during VHSV evolution process were analyzed. The different mutations in European strains and organized glycosylation sites in North American strains were predicted in the G protein. Interestingly, the Asian strains lacked a potential glycosylation site at 459 aa compared to North American strains, which clustered into a large clade including Asian strains. Thus, the genetic distances of individual genes and the mutation of the glycosylation site in the G protein generally conform to the evolutionary trend of complete genomes of VHSV and are also region-specific.

Moreover, the potential recombination events were only detected in an early Japanese strain (KRRV9822). Interestingly, the major and minor potential recombination regions were detected from three later strains which were isolated in 2000, 2008 and 2024. Considering that the KRRV9822 strain was isolated in 1998, the three later strains may not be actual parental strains in those recombination events. Additionally, the potential recombinant strain KRRV9822 was located at the same clade with three potential parental strains in the ML phylogenetic tree. Thus, we inferred that the predicted parental strains are genetically similar to an unsampled ancestral virus involved in earlier recombination events. These strains may represent a lineage that is genetically similar to the unsampled ancestral VHSV strains involved in those earlier recombination events. Furthermore, the potential recombination events were detected in the KRRV9822 strain only. The sparsity of this signal raises the possibility that it does not represent biological recombination. Those potential recombination regions may arise from alignment artifacts, sequencing errors, or stochastic phylogenetic incongruence.

Although all publicly available whole genome sequences of VHSV were incorporated, potential sampling bias inherent to public sequence repositories cannot be fully excluded. The sequence deposition in public databases is largely conditioned by surveillance intensity, regional sequencing capacity, sampling priorities, and research interests. The strains from regions with limited genomic surveillance and asymptomatic or subclinical infections may be under-represented in the dataset. In addition, information on genotypes of partial VHSV strains included in this study is missing in the GenBank database. Accordingly, our findings reflect only the diversity of currently deposited VHSV genome sequences.

Overall, the region-specific genetic relationship and mutation characteristics in genomic nucleic acid and proteins of currently deposited VSHV strains were revealed through genomic sequence analysis and the prediction of key characteristics in this study.

## Figures and Tables

**Figure 1:**
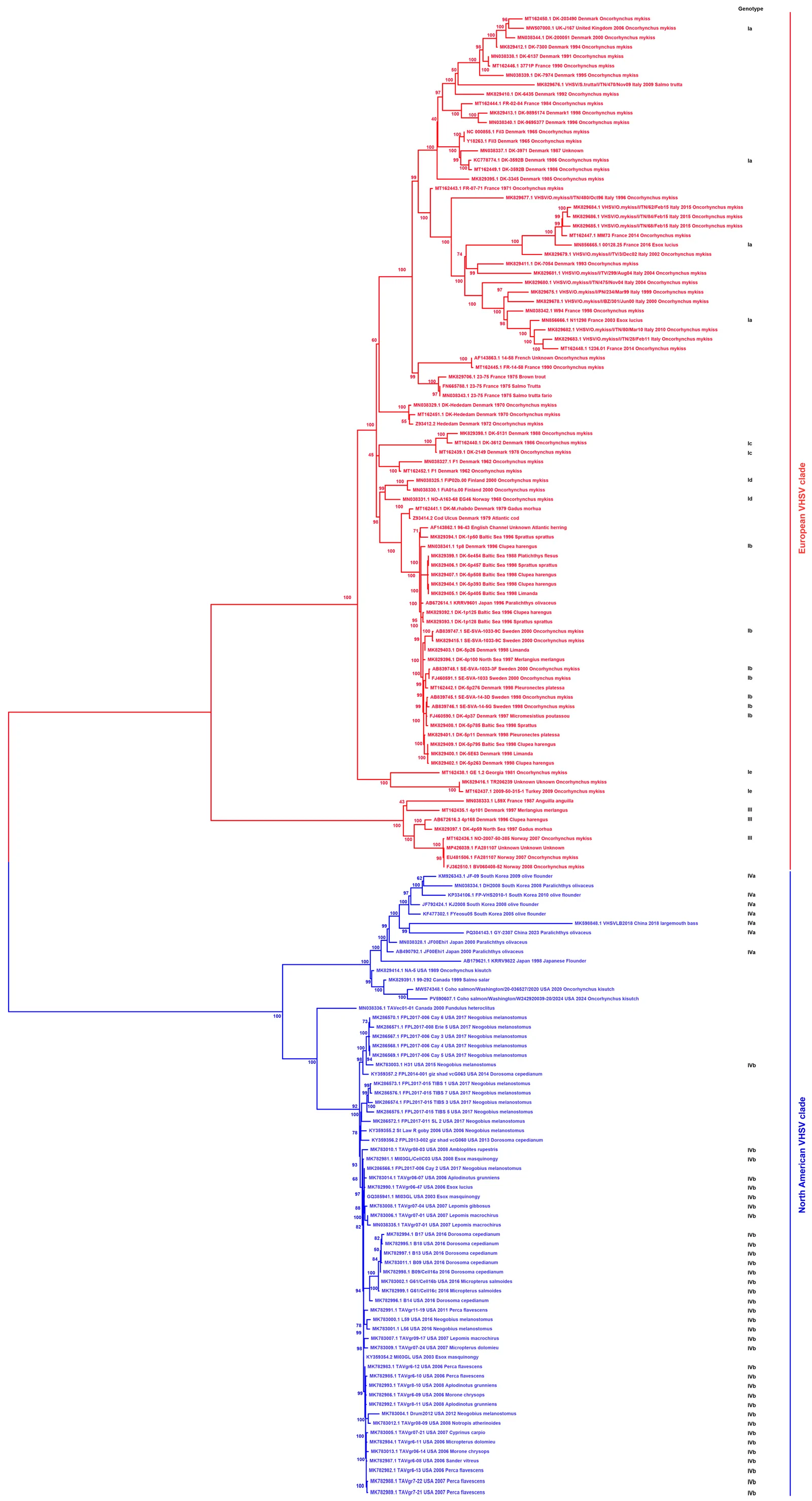
The maximum likelihood phylogenetic tree of global VHSV strains based on their complete genome sequences. The VHSV strains were named based on their accession number, strain/isolation, location, year, and host.

**Figure 2:**
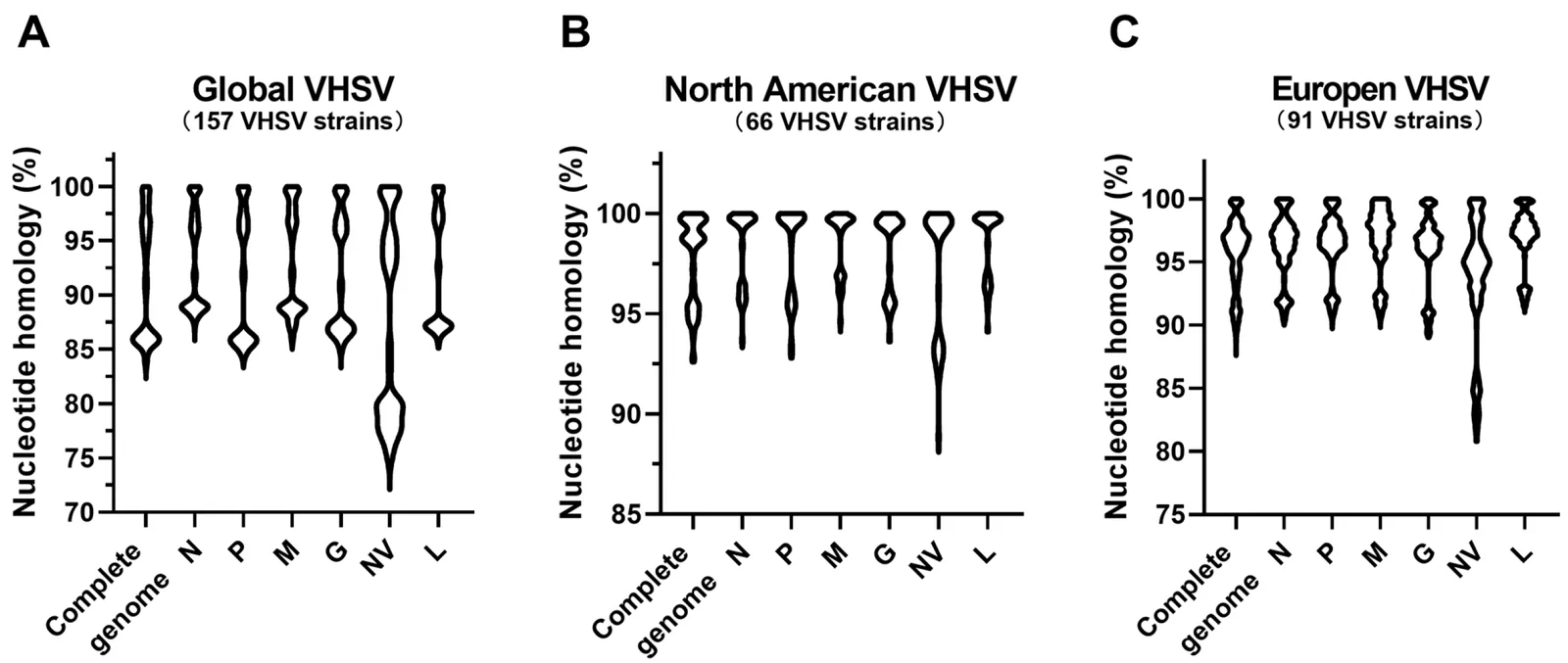
The nucleotide homology of the complete genome of VHSV and different genes. (**A**) Global VHSV strains. (**B**) North American VHSV strains. (**C**) European VHSV strains.

**Figure 3:**
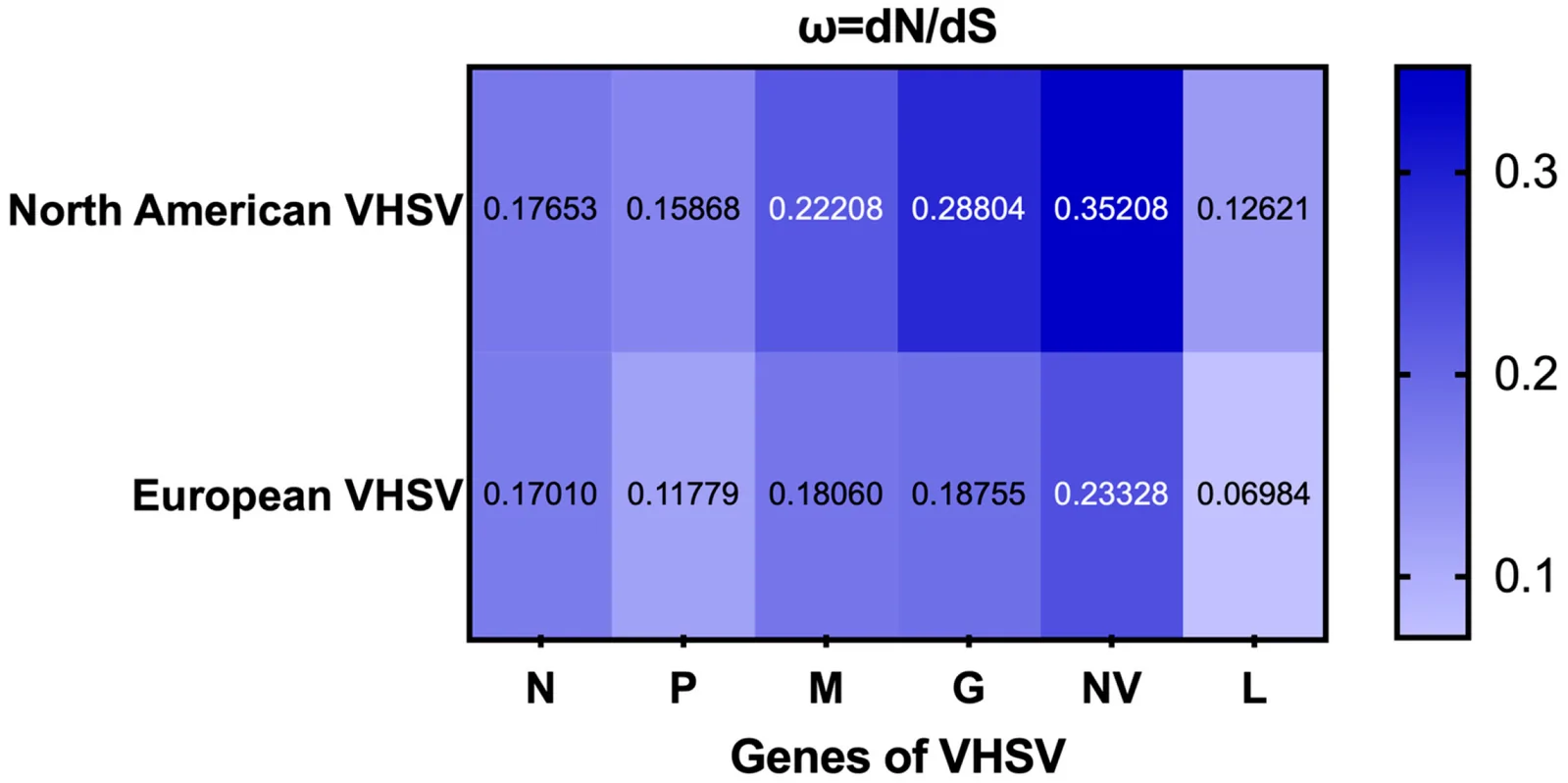
The selection pressure analysis of different genes from North American and European VHSV strains.

**Figure 4:**
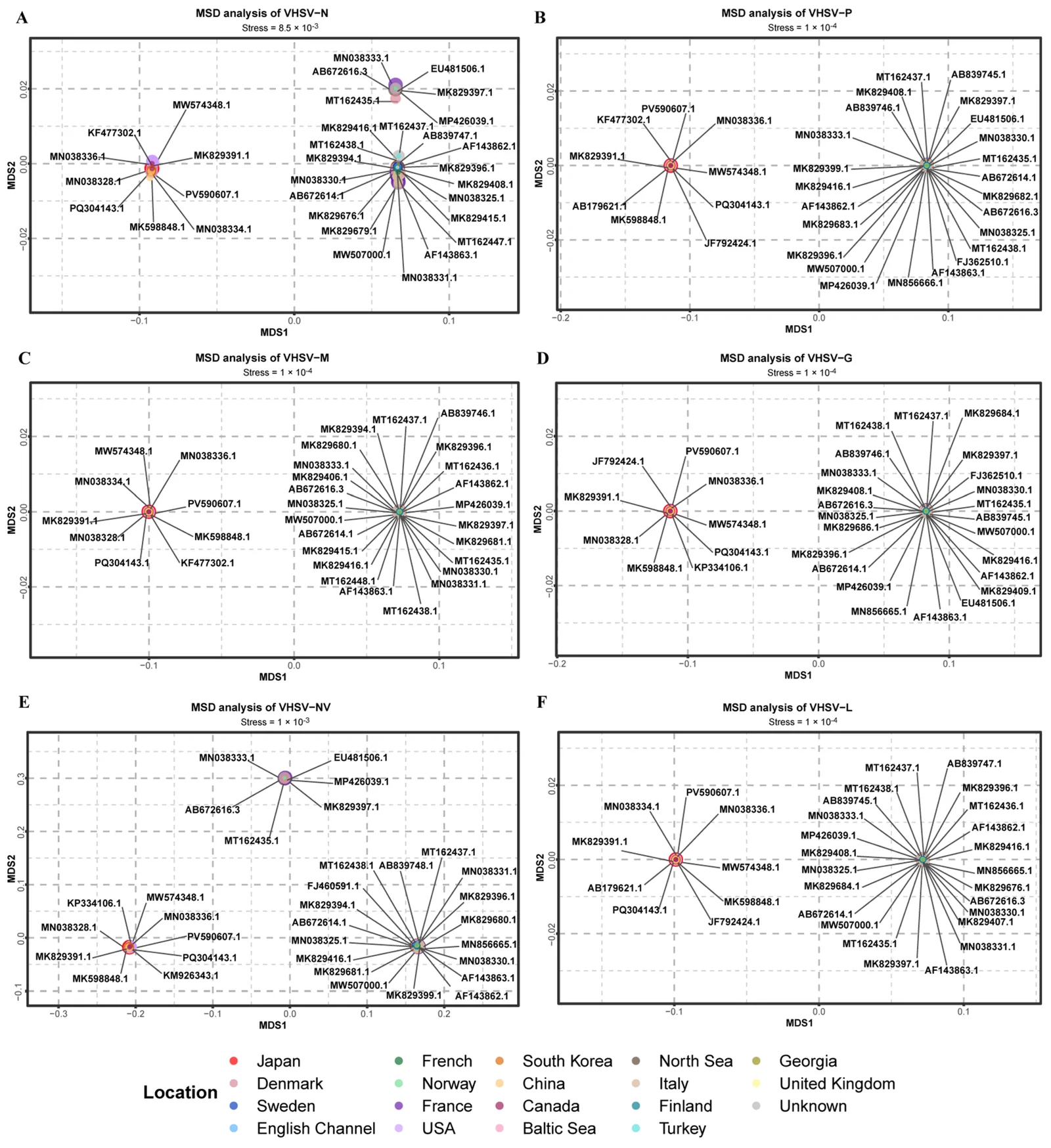
The MSD analysis of VHSV N (**A**), P (**B**), M (**C**), G (**D**), NV (**E**), and L (**F**) proteins based on the K80 model. The different colors represent the locations of VHSV strains. The representative two strains of each location with highest dispersion are labeled with their accession numbers.

**Figure 5:**
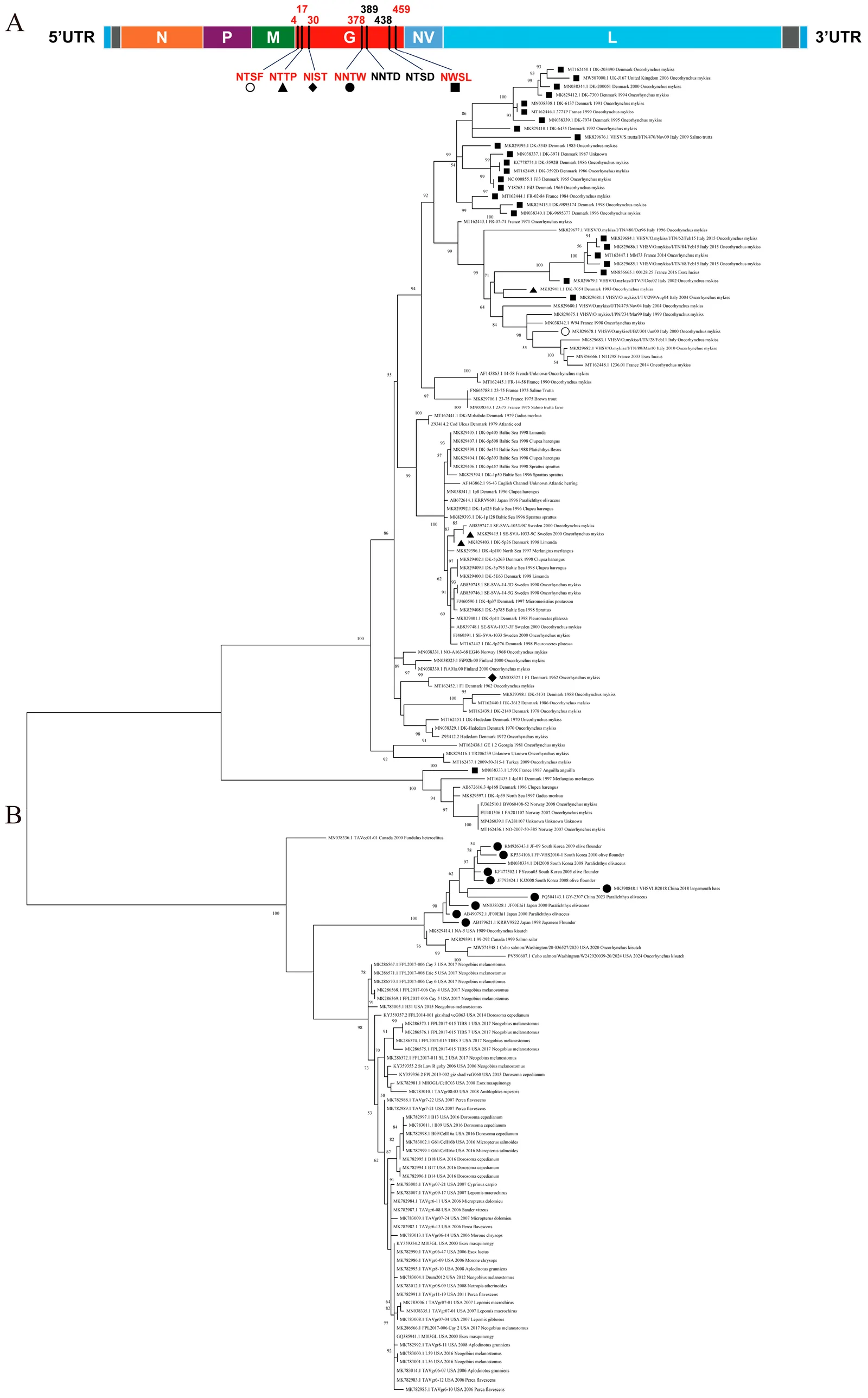
Potential glycosylation sites in the G protein and their distribution in the ML phylogenetic tree based on the G gene. (**A**) The distribution of potential glycosylation sites in the G protein. The red font indicates non-conservative glycosylation sites. The black font indicates conservative glycosylation sites. (**B**) The distribution of non-conservative glycosylation sites in the ML phylogenetic tree of the G gene.

**Figure 6:**
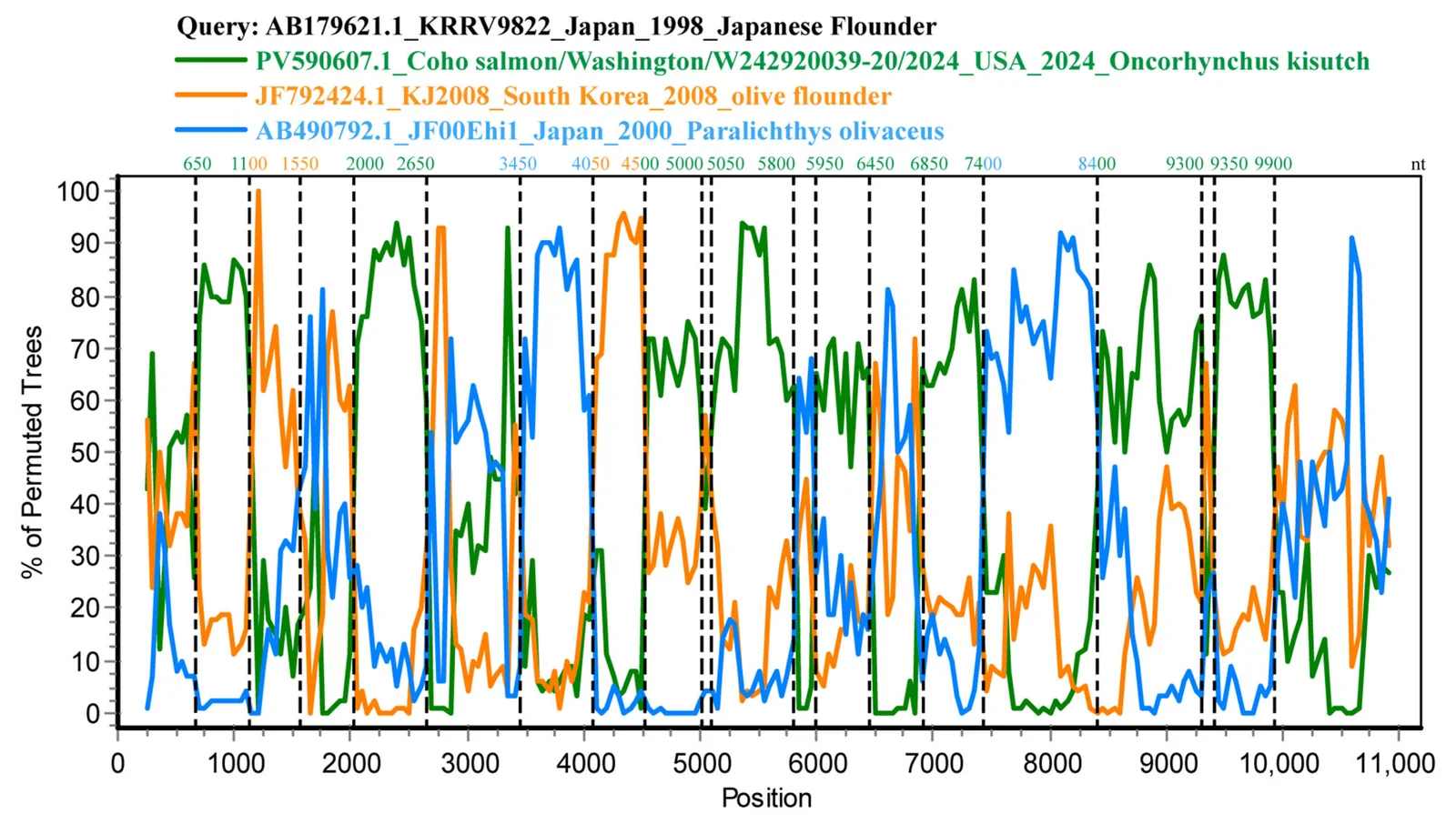
The verification of recombination events in the KRRV9822 strain using Simplot 3.5.1 software. The recombination regions from different strains are shown with different colored dotted lines. Parameters: window size of 500 bp, step size of 50 bp, GapStrip On, Kimura (2-parameter), T/t 2.0, neighbor-joining method.

**Table 1:** The detection of recombination events in the KRRV9822 strain with different algorithm methods.

Major Parental Strain	Algorithm Methods and *p* Value						Minor Parental Strain
	Bootscan	3seq	PhylPro	Maxchi	SiScan	Chimaera	
Coho salmon/Washington/W242920039-20/2024	2.576 × 10^−3^	NS	7.972 × 10^−5^	1.640 × 10^−5^	5.581 × 10^−7^	3.226 × 10^−6^	JF00Ehi1
KJ2008	9.257 × 10^−10^	NS	6.965 × 10^−11^	5.290 × 10^−6^	NS	2.925 × 10^−4^	Unknown

Note. NS = does not support reorganization events.

## Data Availability

The original contributions presented in the study are included in the article/Supplementary Material, further inquiries can be directed to the corresponding author.

## Supplementary Materials

The following supporting information can be downloaded at: https://www.mdpi.com/article/10.3390/microorganisms14102146/s1, Table S1: Global VHSV Strains. Table S2: North American VHSV Strains. Table S3: European VHSV Strains.
